# Building an Australasian paramedicine research agenda: a narrative review

**DOI:** 10.1186/s12961-015-0065-0

**Published:** 2015-12-15

**Authors:** Peter O’Meara, Brian Maguire, Paul Jennings, Paul Simpson

**Affiliations:** La Trobe University, Flora Hill, Victoria Australia; Central Queensland University, Rockhampton Queensland, Australia; Monash University, Melbourne, Australia; University of Western Sydney, Sydney, Australia

## Abstract

The need for paramedicine research has been recognised internationally through efforts to develop out-of-hospital research agendas in several developed countries. Australasia has a substantial paramedicine research capacity compared to the discipline internationally and is well positioned as a potential leader in the drive towards evidence-based policy and practice in paramedicine. Our objective was to draw on international experiences to identify and recommend the best methodological approach that should be employed to develop an Australasian paramedicine research agenda. A search and critical appraisal process was employed to produce an overview of the literature related to the development of paramedicine research agendas throughout the world. Based on these international experiences, and our own analysis of the Australasian context, we recommend that a mixed methods approach be used to develop an inclusive Australasian Paramedicine Research Agenda. This approach will capture the views and interests of a wide range of expert stakeholders through multiple data collection strategies, including interviews, roundtable discussions and an online Delphi consensus survey. Paramedic researchers and industry leaders have the opportunity to use this multidisciplinary process of inquiry to develop a paramedicine research agenda that will provide a framework for the development of a culture of open evaluation, innovation and improvement. This research agenda would assess the progress of paramedicine research in Australia and New Zealand, map the research capacity of the paramedicine discipline, paramedic services, universities and professional organisations, identify current strengths and opportunities, make recommendations to capitalize on opportunities, and identify research priorities. Success will depend on ensuring the participation of a representative sample of expert stakeholders, fostering an open and collaborative roundtable discussion, and adhering to a predefined approach to measure consensus on each topic.

## Background

The delivery of out-of-hospital care in Australia and New Zealand is characterised by strongly centralised and comparatively well-funded paramedic services, with most emergency paramedic services delivered through national, state, territory and regional providers as integral parts of national health systems. An estimated 15,000 paramedics respond to over three million emergency medical calls per year in Australia and New Zealand; the emergency ambulance agencies have a combined annual budget of almost 3 billion Australian dollars [1,2]. Paramedics, and the publicly-funded organisations that employ them, are crucial components of the medical, public health, community health, public safety and disaster response systems.

An increasing number of private paramedic services focussing primarily on either non-emergency patient transport service delivery or on-site medical and primary healthcare, commonly within the industrial-resource, sporting and entertainment domains, are supplementing these government supported services. Additionally, a significant number of paramedics are employed within the respective defence forces of each country [3]. Two professional bodies, Paramedics Australasia and the Australian and New Zealand College of Paramedicine, represent the professional interests of paramedics, while the Council of Ambulance Authorities represents the publicly funded emergency paramedic services in both Australia and New Zealand [4].

The education and training of the paramedicine workforce is in the final stages of its transition from ‘in-house’ vocational training programs to university-level education programs. At least 16 of Australia’s 39 universities now offer entry-level paramedicine programs, while two degree-level programs are offered in New Zealand [5]. It is reported that 5,871 bachelor’s degree paramedicine students were enrolled in Australian universities during 2013 [6]. An estimated 40% of the paramedicine workforce in Australia and New Zealand are now educated to at least a bachelors-degree level, with this proportion growing each year as ‘in-house’ training programs are progressively phased out and vocationally trained paramedics retire or leave the industry for other reasons [7].

## Why paramedicine research?

Research is essential to ensure that the best possible patient care is provided in the out-of-hospital setting and to facilitate the continued development of paramedicine as a health profession [8]. The need for paramedicine research has been recognised internationally through efforts to develop out-of-hospital research agendas in Australia, Europe and North America (Table 1) [9–17]. One of the major advances in Australia and New Zealand has been the development of a relatively large number of paramedics with research degrees – there are now thought to be more than 20 paramedics in Australasia with PhDs and a similar number of doctoral candidates; however, little is known about their employment, aspirations or motivations. This research capacity appears to be strong and robust compared to the discipline internationally, positioning Australasia as a potential leader in the drive towards evidence-based policy and practice in paramedicine.

**Table 1:** **Summary of the development of paramedicine research agendas internationally**

**Research agenda**	**Origin**	**Methodology**	**Developmental lead**
Delbridge et al., 1998 [11]	United States	Expert panel discussions and peer review	Clinicians and researchers
U.S. National Highway Traffic Safety Administration, 2001 [12]	United States	Expert panel discussions and peer review	Government department
Tippett et al., 2003 [9]	Australia	Developmental process identifying barriers to pre-hospital research	Clinicians and researchers
Sayre et al., 2005 [10]	United States	Delphi technique	Government department
Snooks et al., 2008 [27]	United Kingdom	Delphi and rapid scoping techniques	Wide range of health system managers, clinicians and researchers
Snooks et al., 2009 [24]			
O’Donnell & O’Reilly, 2008 [15]	Ireland	Developmental process identifying barriers to pre-hospital research	Clinicians and researchers
Fevang et al., 2011 [14]	Norway	Expert panel of physicians	Pre-hospital physicians
Jensen et al., 2012 [16]	Canada	Delphi technique	Pre-hospital clinicians and researchers, including paramedicine researchers
Patterson and Patterson, 2013 [17]	United States	Facilitated forum of stakeholders and application of the Interview Design Process	Participants in a national consensus conference on community paramedicine

Improvements in service delivery, data collection, paramedicine education and research capacity raise questions about how to prioritise and undertake paramedicine research in a manner that will result in improved patient care and system performance. Following the development of an Australian out-of-hospital service research agenda more than a decade ago [9], the amount and quality of paramedicine research in Australia has increased and has made some impact on the continuing improvement of outcomes. However, as noted at the time, “*… building a strong body of evidence for pre-hospital practice will require an ongoing, targeted and coordinated research effort, leadership in research excellence and a concerted lobby directed to securing recognition of the importance of pre-hospital care to patient outcomes and wider health system performance*” [9].

Out-of-hospital research has led to substantial changes in clinical practice. An example is the discontinuation in the use of pneumatic anti-shock garments (MAST) which had been used since the early 1970’s to increase venous return to the heart in patients with haemorrhagic shock during transport. It was not until 1989, when Mattox et al. [18] published the results of their prospective study showing increased risk of death and longer hospital and intensive care unit length of stays associated with the use of MAST, that the routine use of MAST was stopped [19]. A more recent example of out-of-hospital research changing practice is that of the AVOID study [20], a randomised controlled trial of oxygen therapy versus air in acute myocardial infarction, which challenged the long held belief that oxygen therapy was beneficial in patients experiencing ST-elevation myocardial infarction, and showed that, at 6 months, patients enrolled in this study had an increased infarct size compared with those who did not receive oxygen.

In Australia and New Zealand, there remain questions around research priorities and the overall leadership role of the paramedicine discipline in the production of out-of-hospital research. In addition, current linkages between paramedic services and paramedicine academics should be examined with the goal of developing optimal research outputs. The development of a paramedicine research agenda would make a major contribution toward answering these questions and map the way forward. Using similar arguments as those proposed in the United States [10], an Australasian paramedicine research agenda, or plan, is needed since (1) the consensus process involved in developing a strategic plan for paramedicine research would encourage researchers, the paramedicine profession and paramedic services to work together to develop common research goals; (2) prioritisation of research topics would be useful to funding bodies and researchers; and (3) creating and promoting a common agenda could create a unity of purpose within the paramedicine field and make more effective use of the existing and future research capacity within the profession.

## International efforts to create paramedicine research agendas

Efforts to develop paramedicine research agendas had their genesis in the United States Emergency Medical Services (EMS) Agenda for the Future in 1996 [11,21], the subsequent Agenda for the Future, Implementation Guide [22], and the United States National EMS Research Agenda produced in 2001 [12]. More recently, we have seen similar efforts to establish and implement out-of-hospital research agendas in the United Kingdom [13], Norway [14], Ireland [15], Canada [16] and Australia [9]. In addition, special interest research agendas, such as the Community Paramedicine Research Agenda [17], have emerged.

These international efforts to develop paramedicine research agendas have used variations of the Delphi technique [10,23,24] or expert panels of physicians involved in physician-led EMS systems [14] to gain a consensus on priority issues for research. The United States National EMS Research Agenda was the result of a multidisciplinary process involving expert panel discussions, revision and review by a national writing team, and peer review of the resultant materials [12]. Following this, a strategic plan was created in 2005 identifying priority areas for EMS research in the United States [10].

Until the development of the Canadian Research Agenda for the Future [16], one of the characteristics of efforts to develop research agendas was the limited participation of paramedic researchers in leadership roles. Historically, the main reason given for this has been the dearth of paramedic researchers with the skills and experience to undertake the task of developing a research program [25]. As Snooks et al. [26] stated, “*… the problem of translating research into practice is especially difficult in EMS. Most EMS professionals are not trained to critically evaluate new treatments and so they do not possess the skills to decide whether evidence truly supports their use. … The culture within EMS needs to change to promote research and demand evidence before implementing new system modifications, medications, or drug therapies*”.

The outcomes and conclusions of international research agenda building exercises have reflected the specific environment in which paramedic systems operate. In the United Kingdom, the recommendations from the prioritization exercise were largely concerned with high level policy issues, while in the United States and Norway, more emphasis was given to individual clinical management [10,14,15,24]. In the United Kingdom, the topic receiving the highest priority for research was: “*… the development of new performance measures other than emergency ambulance response times. Other highly ranked priorities included treatment of stroke, cardiac conditions, children and people who self-harm; alternatives to Accident and Emergency treatment; patient information sharing across care providers; access issues; decision support systems; and demand management systems for pre-hospital care*” ([27], p. 2). The research topics identified in the United Kingdom inquiry were wide-ranging and far beyond that examined in other countries. Their report included a rapid scoping review of each topic, which summarised the current research evidence for that topic area, highlighted the knowledge gaps, and made recommendations for future research. For instance, they established that there is no research evidence on how best to involve the public in planning emergency care services [13]. The Norwegian inquiry focused on clinical management issues of relevance to their physician-staffed prehospital system, such as appropriate staffing and training in out-of-hospital critical care and the effect on outcomes, advanced airway management, definition of time windows, the role of out-of-hospital ultrasound, and dispatch criteria for out-of-hospital critical care services [14]. In Ireland, the Centre for Prehospital Research produced a more developmental document that underpins a strategy to develop an out-of-hospital research agenda. Like the United States and Australian efforts before them, they identified obstacles to out-of-hospital research and proposed a set of solutions aimed at establishing a vibrant research culture [15].

The more recent Canadian paramedicine research agenda is much broader than earlier efforts, which reflects on the opportunity to learn from the experiences of other countries that have developed out-of-hospital research agendas. During the development phase of the inquiry, the Canadian team identified four major themes, namely the need for additional research education within EMS; the importance of creating an infrastructure to support pan-Canadian research collaboration; addressing the complexities of involving EMS providers in research; and considerations for a national research agenda [28]. In their final report, the Canadian inquiry team made 19 specific recommendations pertaining to five themes, namely time, opportunities and funding; education and mentorship; culture and collaborations; structure, process and outcome; and EMS research agenda [16]. Their final recommendation focused on the paramedicine research agenda, in which they came to a consensus about potential research topics that require increased or additional research efforts. The topics identified were related to clinical care, health service systems, education, safety, and professional development. They included topics related to the study of time-sensitive interventions, resource utilisation, best practice, measuring competency, and improving both patient and provider safety through system engineering and cultural shifts.

All of these international efforts found similar barriers to paramedicine research as those identified in 2001 by the United States National Highway Traffic Safety Administration, who found that performance of high quality EMS research is hindered by five impediments: paucity of highly skilled researchers; inadequate funding; failure of EMS professionals to understand the importance of conducting EMS research and translating the findings into clinical practice; a lack of integrated information systems that provide for meaningful linkage with patient outcomes; and logistical problems in obtaining informed consent [12].

## Finding a way forward

Clinical care is changing and there is a need to continually evaluate and update paramedic practice. In addition, the rapidly changing needs of society demand a critical evaluation of the out-of-hospital systems’ approaches to day to day emergency responses, the unique needs of an aging community, disaster responses, community health, preventative care, and emerging threats such as pandemics and Ebola-like outbreaks. Existing and emerging models of care need to be investigated and evaluated to determine best practices. There is a need for systems research related to safety, communications, resource deployment and response, workforce planning and integration with other services such as hospitals, public safety, social services and public health services.

The research capacity of the paramedicine discipline in Australia and New Zealand is now extensive. For example, there are a rapidly growing number of PhD-qualified paramedics working within universities and paramedic services. Australian and New Zealand paramedic services have addressed some of the identified barriers to paramedicine research. For example, they have large, sophisticated data collection and management systems that allow data linkages with each other and their respective healthcare systems [7].

Despite these resources, there remain a number of questions around research priorities and the overall leadership role of the paramedicine discipline in the production of research. Building a strong body of paramedicine evidence requires an ongoing, targeted and coordinated research effort, as well as leadership in research excellence and recognition of its importance to patient outcomes and the wider health system performance.

The objectives of developing an Australasian paramedicine research agenda have much in common with other international efforts; these would (1) assess the progress of paramedicine research in Australia and New Zealand since the Australian research agenda was published in 2003; (2) map the research capacity of the paramedicine discipline including paramedic services, universities and professional organisations; (3) identify current strengths and opportunities that may be of benefit to advancing paramedicine research; (4) make recommendations to capitalise on opportunities; and (5) identify Australasian paramedicine research priorities.

Our recommendation for developing an Australasian Paramedicine Research Agenda is that the mixed methods approach used to develop the EMS Research Agenda for Canada in 2011/2012 be used with some modifications, which will account for differences in objectives and the unique Australian and New Zealand paramedicine landscape [16]. The strength of the Canadian approach was its capacity to be inclusive, capturing the views and interests of a wide range of expert stakeholders and other interested parties through multiple data collection strategies, including interviews, roundtable discussions and an online Delphi consensus survey. These culminated in a comprehensive research agenda that has been widely disseminated amongst stakeholders and in the peer-reviewed literature [16,23,28,29].

In Australia and New Zealand, less effort needs to be spent identifying already known barriers to paramedicine research, as a number of them have been successfully addressed. In common with the Canadian inquiry, success will depend on ensuring the participation of a representative sample of expert stakeholders drawn from active out-of-hospital researchers, early career paramedic researchers, paramedics undertaking research degrees, paramedic service research managers and key professionals, industrial and provider organizations, as well as by fostering an open and collaborative roundtable discussion and adhering to a predefined approach to measure consensus on each topic [23].

## Conclusion

It is now time for paramedic researchers to lead a multidisciplinary process of inquiry to develop an Australasian Paramedicine Research Agenda that would make a substantial contribution toward the paramedicine discipline becoming and being recognised as an independent and mature health profession [30]. A research agenda would provide a framework for the development of a culture of innovation and improvement within the industry.

The Agenda would assist research-funding bodies to prioritize research topics, and will unify the field in a way that allows the more effective use of existing and future research capacity within the paramedicine profession. Once an Australasian Paramedicine Research Agenda is developed and disseminated, it would be used to inform budget planning and grant allocations, guide human resource development, and provide arguments to change the way research funding is directed. The ultimate goal of the research agenda will be to develop an out-of-hospital clinical care system that will provide the best possible care using the most effective and efficient delivery methods. This, in turn, will not only reduce death and disability from emergencies, but will also contribute to improvement in medical outcomes, public and community health, public safety, and disaster response across Australia and New Zealand.

## Abbreviations

EMS: Emergency Medical Services
MAST: Pneumatic anti-shock garments
